# 2070 High-intensity focused ultrasound (HIFU) can be used synergistically with tamoxifen to overcome resistance in preclinical and patient derived xenograft models

**DOI:** 10.1017/cts.2018.80

**Published:** 2018-11-21

**Authors:** Rachel Sabol, Hakm Murad, Matthew Burow, Damir Khismatullin, Bruce Bunnell

**Affiliations:** 1 LA CaTS, Tulane University School of Medicine; 2 Tulane School of Science and Engineering; 3 Tulane School of Medicine

## Abstract

OBJECTIVES/SPECIFIC AIMS: The goal of this study is to evaluate a potential strategy to overcome tamoxifen (tam) resistance by using tam in combination with high-intensity focused ultrasound (HIFU). Tam is the most commonly used anti-cancer therapeutic agent in estrogen receptor positive (ER+) breast cancer (BC) which accounts for ~70% of BC cases. Tam treatment decreases a woman’s risk of recurrence by 50%; however, BC that is initially responsive to tam often develops resistance. METHODS/STUDY POPULATION: HIFU deposits acoustic energy locally to a cancerous region, which induces strong vibrations of molecules inside and outside of the cells. The resulting absorption causes rapid heating and mechanical disruption. This clinically relevant, noninvasive, and nonionizing physical force modality, has been shown to synergistically enhance chemical anticancer therapies. RESULTS/ANTICIPATED RESULTS: In this study we found that treatment of MCF7 cells with HIFU and tam has additive antiproliferative effects and mediates increased cell death. Additionally, we used tam resistant (TR) MCF7 cells that had been exposed to low-dose tam over time until they acquired resistance. When MCF7 TR are treated with tam there is no change in viability; however, treatment with HIFU in combination with tam decreased viability of both MCF7 and MCF7 TR to 19% and the viability of the cell lines was indistinguishable. We next evaluated the effect on MCF7 Y537S mutant ESR1, where ER is mutated to be constitutively active. Treatment of MCF7 Y537S had no significant decrease in viability of combination therapy compared with viability after HIFU alone. Analysis of ERalpha gene expression showed that HIFU treatment increased ERalpha expression in MCF7 TR cells, thus resensitizing these cells to tam and allowing these therapies to work synergistically. Our team developed a system to evaluate the potential of this combination of therapies in a patient-derived xenografts (PDX) model. PDX have emerged as a novel translational tool for cancer research with the potential to more accurately recapitulate the molecular and behavioral aspects of cancer. The WHIM20 PDX is a tamoxifen resistant tumor where the patient developed the Y537S mutation in ESR1. Ex vivo experiments on PDX tumor pieces demonstrated that combination therapy of HIFU and tam work synergistically to increase cell death of these tumors. Further, cryogenic-scanning electron microscopy was utilized to directly demonstrate the physical disruption to both cellular and tumor microenvironment post exposure to combination treatment. DISCUSSION/SIGNIFICANCE OF IMPACT: These studies present a novel translational strategy to overcome tamoxifen resistance in ER+BC.

